# Effects of N_2_ Partial Pressure on Growth, Structure, and Optical Properties of GaN Nanorods Deposited by Liquid-Target Reactive Magnetron Sputter Epitaxy

**DOI:** 10.3390/nano8040223

**Published:** 2018-04-07

**Authors:** Muhammad Junaid, Ching-Lien Hsiao, Yen-Ting Chen, Jun Lu, Justinas Palisaitis, Per Ola Åke Persson, Lars Hultman, Jens Birch

**Affiliations:** Thin Film Physics Division, Department of Physics, Chemistry, and Biology (IFM), Linköping University, SE-581 83 Linköping, Sweden; Muhammad.Junaid@husqvarnagroup.com (M.J.); james_hokkeko@yahoo.com.tw (Y.-T.C.); junlu@ifm.liu.se (J.L.); juspa@ifm.liu.se (J.P.); per.persson@liu.se (P.O.Å.P.); larhu@ifm.liu.se (L.H.)

**Keywords:** GaN, nanorods, X-ray diffraction, TEM, photoluminescence, magnetron sputter epitaxy, sputtering

## Abstract

GaN nanorods, essentially free from crystal defects and exhibiting very sharp band-edge luminescence, have been grown by reactive direct-current magnetron sputter epitaxy onto Si (111) substrates at a low working pressure of 5 mTorr. Upon diluting the reactive N_2_ working gas with a small amount of Ar (0.5 mTorr), we observed an increase in the nanorod aspect ratio from 8 to ~35, a decrease in the average diameter from 74 to 35 nm, and a two-fold increase in nanorod density. With further dilution (Ar = 2.5 mTorr), the aspect ratio decreased to 14, while the diameter increased to 60 nm and the nanorod density increased to a maximum of 2.4 × 10^9^ cm^−2^. Yet, lower N_2_ partial pressures eventually led to the growth of continuous GaN films. The observed morphological dependence on N_2_ partial pressure is explained by a change from N-rich to Ga-rich growth conditions, combined with reduced GaN-poisoning of the Ga-target as the N_2_ gas pressure is reduced. Nanorods grown at 2.5 mTorr N_2_ partial pressure exhibited a high intensity 4 K photoluminescence neutral donor bound exciton transitions (D^0^X_A_) peak at ~3.479 eV with a full-width-at-half-maximum of 1.7 meV. High-resolution transmission electron microscopy corroborated the excellent crystalline quality of the nanorods.

## 1. Introduction

Low-dimensional semiconductor nanostructures, such as nanorods (NR), have drawn an interest due to their great prospects in novel nanotechnology applications as well as in fundamental material physics, i.e., understanding the growth mechanisms of nanostructures, effects of different surface energies on the adatom mobility, etc. These are promising components for future applications in nano-electronics and nano-optoelectronics [1,2,3,4]. GaN, with a direct wide band gap of 3.4 eV and a high electron mobility is a promising material and is being exploited in these kinds of devices. Catalyst-free GaN-NR growth has successfully been demonstrated by chemical vapor deposition (CVD), hydride vapor phase epitaxy (HVPE) [5,6,7], physical vapor deposition (PVD) (i.e., molecular beam epitaxy (MBE) [8,9,10,11]) and direct-current magnetron sputter epitaxy (DC-MSE) [12,13,14,15]. Magnetron sputter deposition is a well-established method which can easily be up-scaled to grow over very large areas for industrial applications. In addition, it provides for low-energy ion assisted deposition which can be used to lower the growth temperature. DC-MSE is a promising method as it employs ultra-high vacuum (UHV) conditions and ultra-high-purity source materials, thus combining the advantages of magnetron sputter deposition and those of MBE. DC-MSE has also been demonstrated for the growth of high quality GaN continuous epilayers [16,17] as well as NRs. Besides the mass and energy of incident reactive species, selective control of metal–ion fluxes, substrate bias synchronized to probe gas–ion or metal–ion irradiation, is available in a more controlled manner with more complicated sputtering configurations being employed, such as high-power impulse magnetron sputtering (HiPIMS), hybrid HiPMS/DC-MSE co-sputtering using synchronized pulsed substrate bias, MSE with applying external magnetic field, etc. [18,19,20,21]. These methods have been applied to achieve single-phase multi-nary alloys grown at low temperature, which demonstrates a high capability to use MSE for the material growth of high-performance optoelectronics. 

DC-MSE of GaN-NRs/Si (111) has been reported, using a pure N_2_ environment, with a total pressure varying from 5 to 20 mTorr. The NRs exhibited excellent optical properties with band-edge (BE) emission at 3.477 eV with a full-width-at-half-maximum (FWHM) value of 1.7 meV when grown at the highest N_2_ partial pressure value of 20 mTorr. This coincided with the best achieved structural quality, highest aspect ratio, and highest growth rate of the NRs. 

However, working at high pressures of pure N_2_ with DC-MSE, such as 20 mTorr, poses several challenges. For example, it is difficult to maintain a stable process at such pressures when sputtering in a N_2_-rich environment. Sputtering a metallic Ga target under such conditions results in the formation of a GaN compound of low electrical conductivity on the surface, so called target poisoning, which is a major issue. Target poisoning is known to strongly limit the achievable deposition rate and, in some cases, inhibit sputtering altogether. Moreover, after the growth of GaN-NRs using a pure nitrogen environment, target poisoning effects make it impossible to grow continuous GaN epilayers, which require an Ar/N_2_ mixture, using the same nitrided target. Another disadvantage of using high working pressures, like 15 or 20 mTorr, is that throttling of the UHV pumping system often is required in order to maintain a stable pressure. Such practice reduces the pumping speed and thus, effectively eliminates the UHV conditions during growth, which increases the probability of impurity incorporation into the growing material. A high process pressure also leads to gas scattering of the sputtered Ga which, in turn, leads to a lower degree of utilization of the source material and unwanted deposition of Ga remote from the substrate. It is thus desirable to lower the total pressure and reduce the amount of N_2_ in the sputtering gas for DC-MSE GaN-NRs growth. 

In this work, we have investigated the effect of a low N_2_ partial pressure in Ar/N_2_ mixture (5 mTorr), compatible with GaN epilayer growth, to achieve high quality GaN-NR growth onto Si substrates at 1000 °C by using the DC-MSE growth process. The dependence of the structural and the optical properties on N_2_ partial pressure is discussed, taking into account the changing growth modes combined with a reduced poisoning effect of the Ga-target. The as-grown nanorods are characterized by scanning electron microscopy (SEM), cross-sectional high-resolution transmission electron microscopy (HRTEM), X-ray diffraction (XRD), and micro-photoluminescence (µ-PL) spectroscopy.

## 2. Materials and Methods

The GaN nanorods were grown by home-made reactive DC-MSE for 120 min on Si(111) substrates kept at a temperature of 1000 °C. A very careful temperature calibration was performed to measure the exact surface temperatures on the substrate, and Minolta Land Cyclops infrared-pyrometers (Ametek Land, Dronfield, United Kingdom) were also used as an extra measurement tool during the calibration process. During growth, we also checked the surface temperature from time to time to reconfirm the surface temperature. 

Liquid Ga (99.99999%), contained in a horizontal stainless-steel tray of 50 mm diameter, was used as a magnetron sputtering target with a constant magnetron power of 10 W. The sputtering was carried out in a reactive environment using N_2_ (99.999999%) and Ar (99.999999%) as working gases. The high-purity of gas (99.999999%) was achieved by using 99.9999% gas, further purified by special gas purifiers. The depositions were performed in a homemade UHV-chamber, without any cooling shroud, with a base pressure of ~5 × 10^−9^ Torr. The partial pressures of N_2_ (*P*_N2_) and Ar (*P*_Ar_) varied from *P*_N2_ = 5 mTorr and *P*_Ar_ = 0 mTorr to *P*_N2_ = 1 mTorr and *P*_Ar_ = 4 mTorr, keeping the total pressure constant at 5 mTorr for all depositions. For convenience, we will most often refer only to the *P*_N2_ values in this article, while *P*_Ar_ + *P*_N2_ always equals 5 mTorr. During growth, no substrate rotation was used. 

The Si (111) substrates were cleaned in ultrasonic baths of trichloroethylene, acetone, and 2-propanol for 5 min at each step and were blown dry with N_2_ just prior to inserting them into the chamber via a load-lock system. 

A Zeiss-Leo 1550 field emission SEM (Zeiss, Oberkochen, Germany), operated at 10 kV, was used to study the surface morphology and to measure the length, diameter, and areal number density of the nanorods from side- and plan-view micrographs of the samples. Growth rate was determined by using the average length of the nanorods, measured by SEM cross-sectional micrograph, and dividing it by the total growth time. The average rod density was determined using topographical and cross-sectional micrograph by SEM and calculating the number of rods in certain area.

The microstructures of the as-deposited GaN nanorods were investigated by cross-sectional HRTEM using a FEI Tecnai G2 TF 20 UT field-emission TEM (Thermo Fisher Scientific, Eindhoven, Nederland), operated at 200 kV. The cross-sectional TEM samples were prepared by mechanical polishing, followed by Ar ion milling at 5 keV. Final polishing was done using low energy ions at 2 keV.

To characterize the crystal structure, overview *θ*–2*θ* XRD scans were performed with a Philips 1820 Bragg-Brentano diffractometer (Malvern, Almelo, Netherlands). A Phillips X’Pert MRD diffractometer (Malvern, Almelo, Netherlands), using Cu Kα radiation, with a four-axis goniometer, and configured with 1 × 1 mm^2^ crossed slits as the primary optics and a 0.27° parallel plate collimator as secondary optics was used for pole-figure measurements. Pole-figure measurements were performed for the GaN (10$\overset{-}{15}$)-plane spacing with a fixed 2*θ* angle of ~105.3°.

Optical properties were characterized by μ-PL (homemade) at 4 K in a backscattering geometry. A continuous-wave (CW) Coherent Verdi/MBD-266 laser system (λ_exc_ = 266 nm) was used as the excitation source. The setup details can be found in ref. [22].

## 3. Results and Discussion

**Figure 1:**
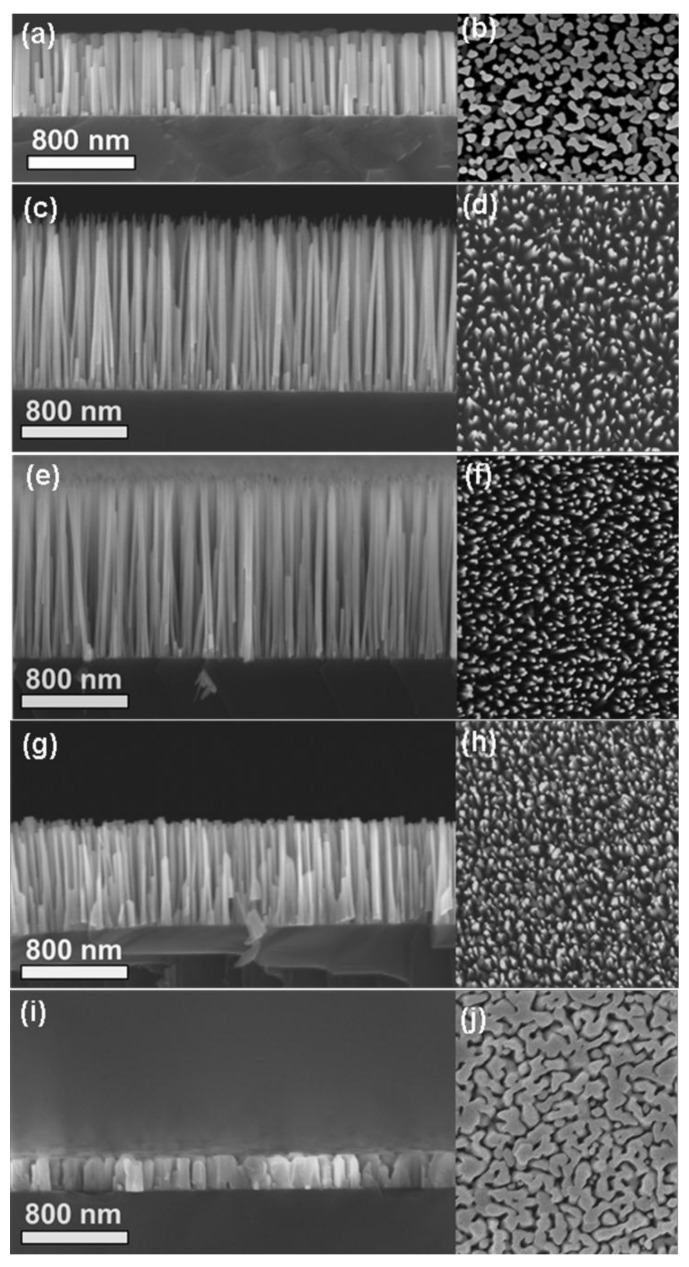
SEM micrographs showing side views (**left**) and top views (**right**) from GaN nanorod samples grown at (**a**,**b**) *P*_N2_ = 5 mTorr; (**c**,**d**) *P*_N2_ = 4.5 mTorr; (**e**,**f**) *P*_N2_ = 3.5 mTorr; (**g**,**h**) *P*_N2_ = 2.5 mTorr; (**i**,**j**) *P*_N2_ = 1 mTorr pressure on Si(111) substrates.

Figure 1a–j show side- and plan-view SEM images of a sample with self-assembled GaN nanorods grown onto Si (111) substrates at different *P*_N2_ and *P*_Ar_, with a constant total pressure at 5 mTorr. Figure 1a,b present the cross-sectional and top-view morphologies of the rods grown in a pure nitrogen atmosphere (*P*_N2_ = 5 mTorr). The cross-sectional image shows that the nanorods have a uniform length of ~550 nm and an average diameter of ~74 nm. The top-view morphology image shows that the rods exhibit highly irregular cross-sections. Figure 1c,d show the cross-section and top-view of the NRs grown at *P*_N2_ = 4.5 mTorr, respectively. This reveals that exchanging a small amount of N_2_ to Ar (*P*_Ar_ = 0.5 mTorr) has a dramatic effect on the length and diameter of the rods. The average diameter of the rods shrinks to ~34 nm, and the average length increases to ~1260 nm. The rods are now well-separated without any coalescence, as shown in Figure 1d. Further dilution of the N_2_ working gas with Ar causes the rods to shrink in length, with average values of 1190 nm and 820 nm for *P*_N2_ = 3.5 and 2.5 mTorr, respectively (see Figure 1e,g), while the average diameter increases to 36 nm and 60 nm for *P*_N2_ = 3.5 and 2.5 mTorr, respectively. For the *P*_N2_ of 2.5 mTorr, the bases of some of the rods are slightly broader with a shoulder, after which the rods become narrower in diameter, similar to what frequently has been observed in MBE grown GaN-NRs on Si(111) [23,24]. The average diameter of the base is ~80 nm, and the top part is ~40 nm. Decreasing the P_N2_ beyond 2.5 mTorr leads to the formation of a continuous, 245 nm thick, GaN film with large columnar grains with an average width of ~100 nm, instead of forming well separated NRs, as seen in Figure 1i,j.

**Figure 2:**
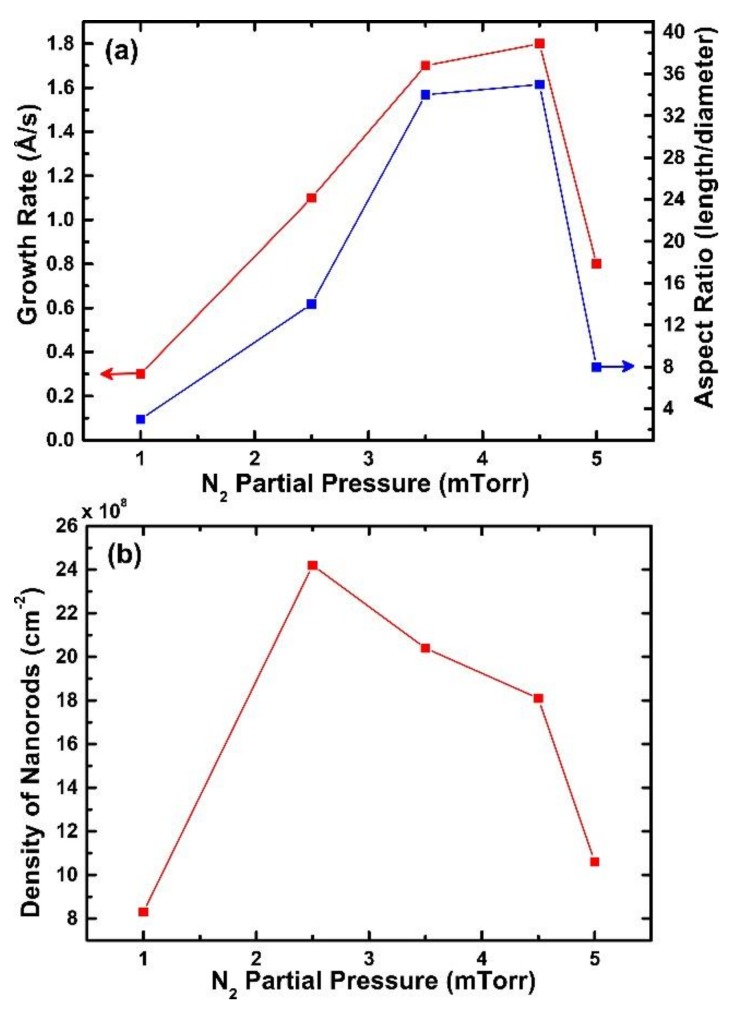
(**a**) Growth rate and aspect ratio and (**b**) variation of density of rods with nitrogen partial pressure, where N_2_ partial pressures varying from 5 to 1 mTorr.

The dependence of N_2_ partial pressure versus the growth rate and aspect ratio for the GaN-NRs extracted from Figure 1 is represented in Figure 2a. Upon introducing a small amount of Ar (*P*_N2_ = 4.5 mTorr and *P*_Ar_ = 0.5 mTorr) into the working gas, the GaN NRs growth rate is increased by a factor of two to 1.8 Å/s, and the aspect ratio increases from 8 to ~35 compared to the pure N_2_ case. Morphologically, these NRs are very similar to the GaN-NRs grown at 20 mTorr partial pressure in a pure N_2_ environment. By further diluting the N_2_ gas to a *P*_N2_ of 2.5 mTorr, the growth rate and aspect ratio continuously decrease to 1.1 Å/s and ~14, respectively. 

The estimated density of the nanorods presented in Figure 2b shows that the rod density increases as Ar is introduced into the sputtering gas. The highest density is obtained at *P*_N2_ = 2.5 mTorr, whereafter, the density drops again.

We suggest the following model to explain the observed changes in the evolution of the NR morphology as the gas composition is changed: When sputtering in a pure N_2_ environment, the Ga-target assumes a poisoned state, which reduces the sputtering yield of Ga. Moreover, the pure N_2_ atmosphere means that GaN nucleation occurs in a N_2_-rich regime. Such conditions are known to severely limit the Ga adatom mobility [25,26,27,28,29,30] and lead to small GaN nuclei which determines the diameter of the GaN nanorods, as also commonly seen in self-induced GaN NRs grown under nitrogen-rich conditions by plasma-assisted MBE [30,31,32]. These nuclei are formed with a lower verticality along the *c*-axis orientation of the Si substrate with a native amorphous SiO*x* during incubation, which has been observed in selective-area growth cases [14,15]. After the nuclei are formed, vertical growth of GaN nanorods can be explained on the basis of the so-called surface diffusion induced growth mechanism, which elucidates the effect of Ga adatom diffusion on the base, apex, and side walls of the nanorods as well as the incoming flux effect. During this MSE growth, the incoming flux is normally incident to the substrate, which minimizes the shadowing effect and fosters the growth along the flux direction. Since the growth along the *c* axis is faster than other orientations and can be further enhanced by surface diffusion at higher growth temperatures, the verticality of the NRs becomes higher after the nucleation stage. This model has successfully described the growth of GaN nanorods in MSE, which is very similar to MBE-grown GaN NRs. 

By introducing Ar into the working gas, the Ga sputter yield increases, both due to better momentum transfer from the sputter gas (because of the larger mass of Ar) and also due to reduced target poisoning [33,34]. Thus, by increasing the *P*_Ar_ to 0.5 mTorr, the Ga flux will increase compared to the case of pure N_2_. At the same time, the amount of the N available at the substrate surface for GaN formation will not be changed significantly because of the overall high N super saturation (N-rich conditions), such that the Ga limited adatom mobility condition prevails [25,31,32,35]. This will lead to a higher nucleation density (i.e., smaller nuclei), which, in turn, leads to an increased density of narrower and longer NRs, as is observed in our data. The continued increase in NR density as *P*_N2_ is decreased to 2.5 mTorr shows that the nucleation in this pressure range essentially occurs in a N-rich regime (where the Ga adatom mobility is low) with an increasing Ga-flux. However, at the same time, the reduced aspect ratio and growth rate observed upon further dilution of the N_2_ sputter gas show that the conditions are changed such that the surface diffusion induced growth mechanism is less favored. This is an indication that the growth conditions are changing to less N-rich conditions, which increases the possibility of forming bigger GaN nuclei and the base of mature NRs, as reflected by a reduction in the aspect ratio and density of NRs (see Figure 2). At some point, upon further dilution of the N_2_ gas, the target surface will change from being GaN-poisoned to metallic, which will lead to a strong increase in the Ga flux towards the substrate, possibly leading to a fast transition into N-deficient conditions. At the high growth temperature of 1000 °C, the desorption rate of Ga adatoms from the substrate will increase if the availability of N_2_ becomes limited. Thus, the amount of Ga on the substrate will decrease at the same time as the Ga adatom diffusion length increases, which leads to a lower nucleation density and growth of larger grains at a low growth rate. Based on the observed transition from NR growth to the formation of a continuous GaN film (made up by large grains, see Figure 1i) at a low growth rate, when going to *P*_N2_ = 1 mTorr, we propose that target poisoning ceases and the growth changes to N-deficient conditions at an N_2_ pressure between 1 mTorr and 2.5 mTorr. Thus, we conclude that the morphology of DC MSE grown GaN-NRs is qualitatively influenced by the Ga and N fluxes in a similar way as MBE-grown GaN NRs [25,31,32].

**Figure 3:**
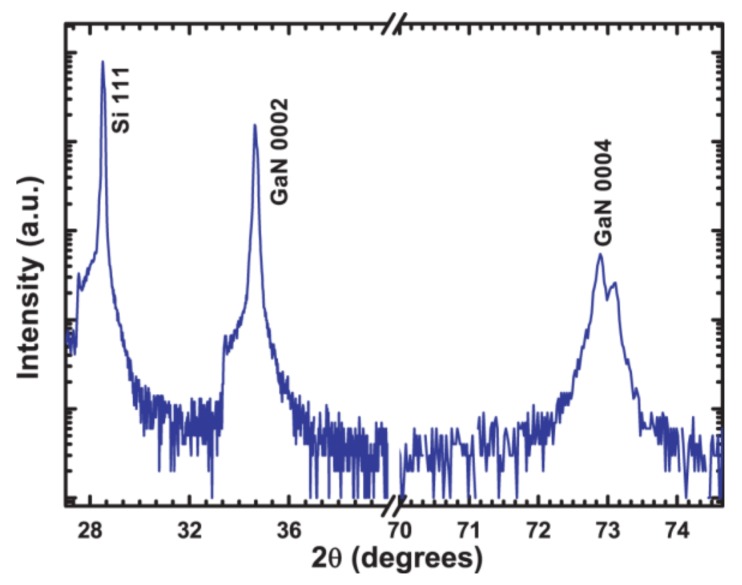
XRD *θ*/2*θ* scans from the sample grown at *P*_N2_ = 4.5 mTorr.

**Figure 4:**
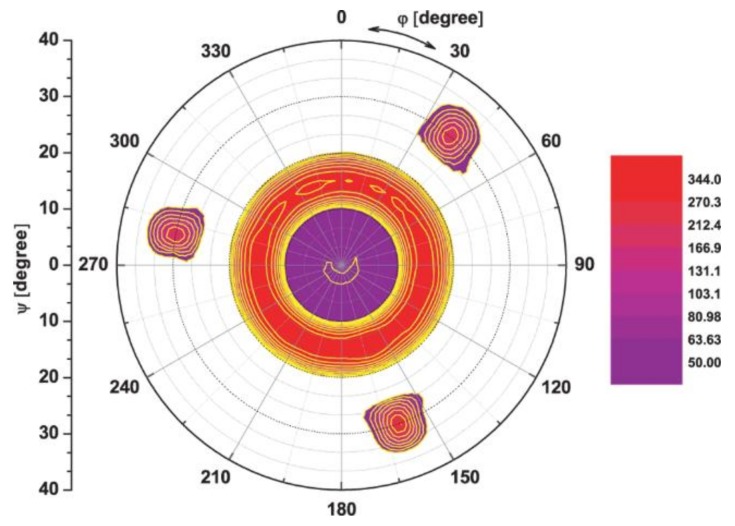
10$\overset{-}{15}$ Pole-figure measurements of GaN nanorods grown at *P*_N2_ = 4.5 mTorr.

Long range XRD *θ*/2*θ* measurements were performed for the samples grown at different partial pressures and all the measurements were similar, showing only GaN 0002 and 0004 reflections along with the Si substrate peak. An example is shown in Figure 3, which shows an XRD *θ*/2*θ* scan from the sample grown at *P*_N2_ = 4.5 mTorr. Thus, the XRD results show that all GaN-NRs are oriented relative to the *c*-axis along the growth direction. To determine the in-plane orientation of the GaN nanorods, pole figures were recorded from all samples. A representative {10$\overset{-}{15}$} pole figure (from *P*_N2_ = 4.5 mTorr sample) is shown in Figure 4. Similar results were observed from all samples. The pole figure reveals a high intensity ring at *ψ* ~20°, corresponding to an azimuthally random orientation of the {10$\overset{-}{15}$} planes, i.e., the nanorods are oriented with the *c*-axis along the growth direction with a random in-plane orientation. In Figure 4 three reflections are also visible at *ψ*~35°, separated by ~120° in *φ*, which correspond to the three-fold symmetry of the {440} planes of (111)-oriented Si.

**Figure 5:**
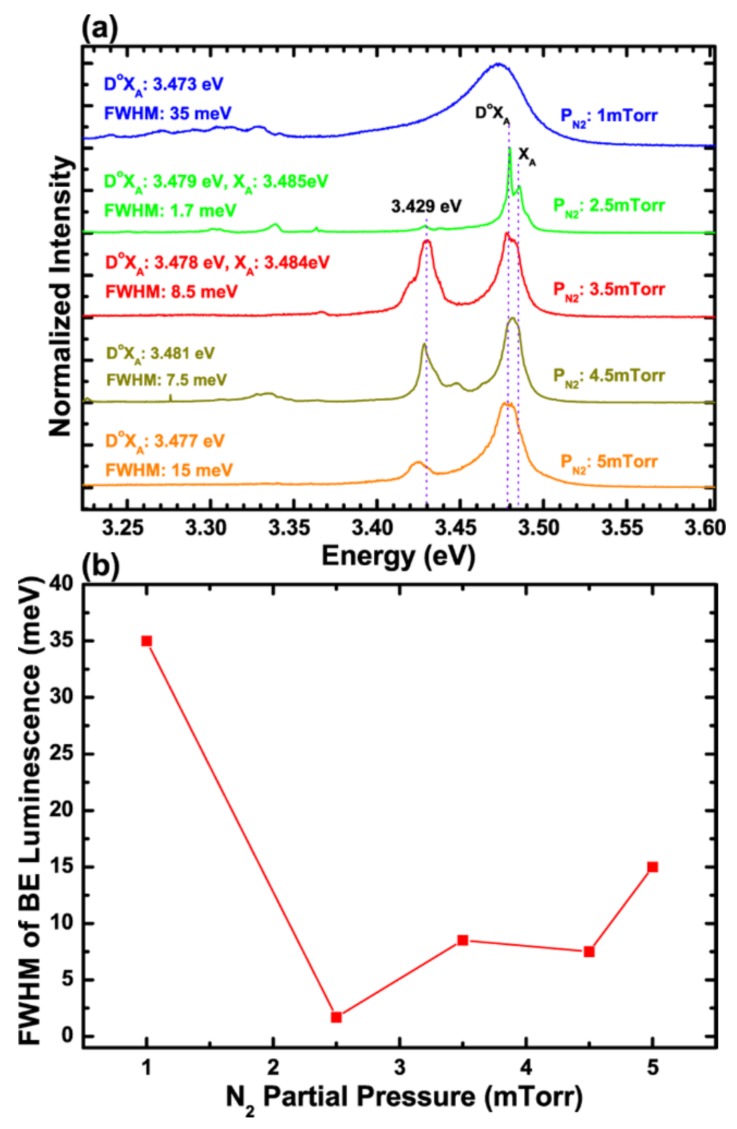
(**a**) µ-PL Spectra from GaN nanorods grown at different nitrogen partial pressures and (**b**) full-width-at-half-maximum (FWHM) of band-edge luminescence vs nitrogen partial pressure.

Low-temperature µ-PL spectra characterizing the optical properties of the GaN-NRs are shown in Figure 5a. In the spectra, peaks from GaN have been identified as the free A exciton recombination (X_A_) and neutral donor bound exciton transitions (D^0^X_A_) [36], also known as the band-edge (BE) luminescence. For the samples grown at *P*_N2_ = 5, 4.5, 3.5, 2.5 and 1 mTorr, the D^0^X_A_ peak is visible at ~3.477, 3.481, 3.478, 3.479, and 3.473 eV, respectively. For the *P*_N2_ = 3.5 mTorr and 2.5 mTorr samples, the X_A_ peak is also well separated and visible at 3.484 and 3.485 eV, respectively. The corresponding FWHMs of the D^0^X_A_ peaks are shown in Figure 5b. It can be seen that an exceptionally narrow emission line of only 1.7 meV width is produced by the NRs grown at *P*_N2_ = 2.5 mTorr. The narrow BE luminescence from the GaN-NRs is attributed to high crystalline quality and low impurity incorporation. In fact, the NRs grown at *P*_N2_ = 2.5 mTorr show very similar optical properties as those previously demonstrated by the MBE-grown GaN NRs and by DC-MSE-grown GaN NRs grown in a pure N_2_ atmosphere at 20 mTorr. Those NRs were shown to be almost entirely free from structural defects. The peaks visible at ~3.429 eV for all the NRs grown in this study at different partial pressures of Ar and N_2_ are attributed to basal plane stacking faults (SFs) as reported in ref. [37,38,39]. In addition, we also observed very broad yellow luminescence (YL) centered at around 2.35 eV (not shown here). The peak is referred to the structural defect and/impurity incorporation in GaN [40,41,42]. The peak intensity of YL is four times weaker than the D^0^XA emissions in all samples and has a similar trend of stacking-fault emissions with varying partial pressure. Hence, it is more relevant to the generation of structural defect (plasma-induced defects) in the NRs. However, impurity incorporation through diffusion into structural defects, either during growth or after sample’s exposure to air, cannot be ruled out completely. Such defect-related peaks were not observed in the spectra from the DC-MSE GaN nanorods grown at a working pressure of 20 mTorr in a pure N_2_ atmosphere [12]. However, at lower pressures, such as in the present work, the plasma surface interaction increases significantly due to the increased mean free path in the plasma, which increases the local heat, so that crystal damage, caused by the energetic species impinging on the surface of the growing crystal, may occur. 

**Figure 6:**
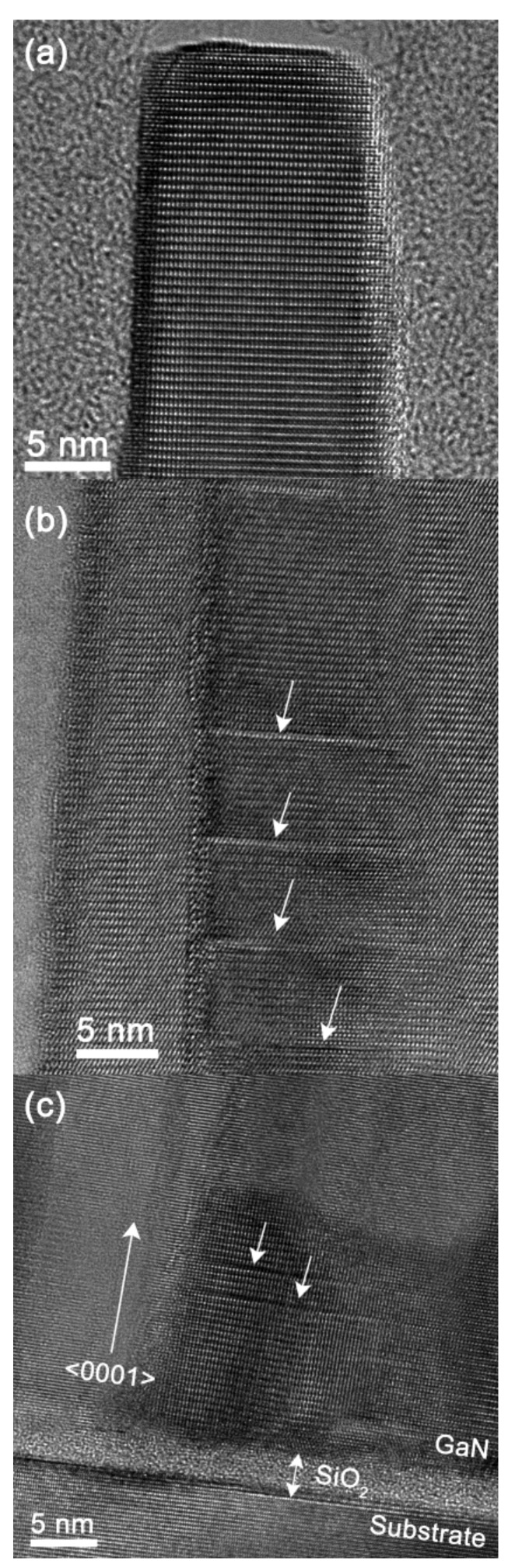
Cross-sectional HRTEM images from a GaN nanorod grown at *P*_N2_ = 4.5 mTorr on Si(111) substrate showing (**a**) the top part of the nanorod; (**b**) the middle part of the nanorod with stacking faults indicated by the arrows; and (**c**) the interface between the substrate and nanorods, containing the native oxide layer at the interface. Stacking faults close to the interface are indicated by arrows.

**Figure 7:**
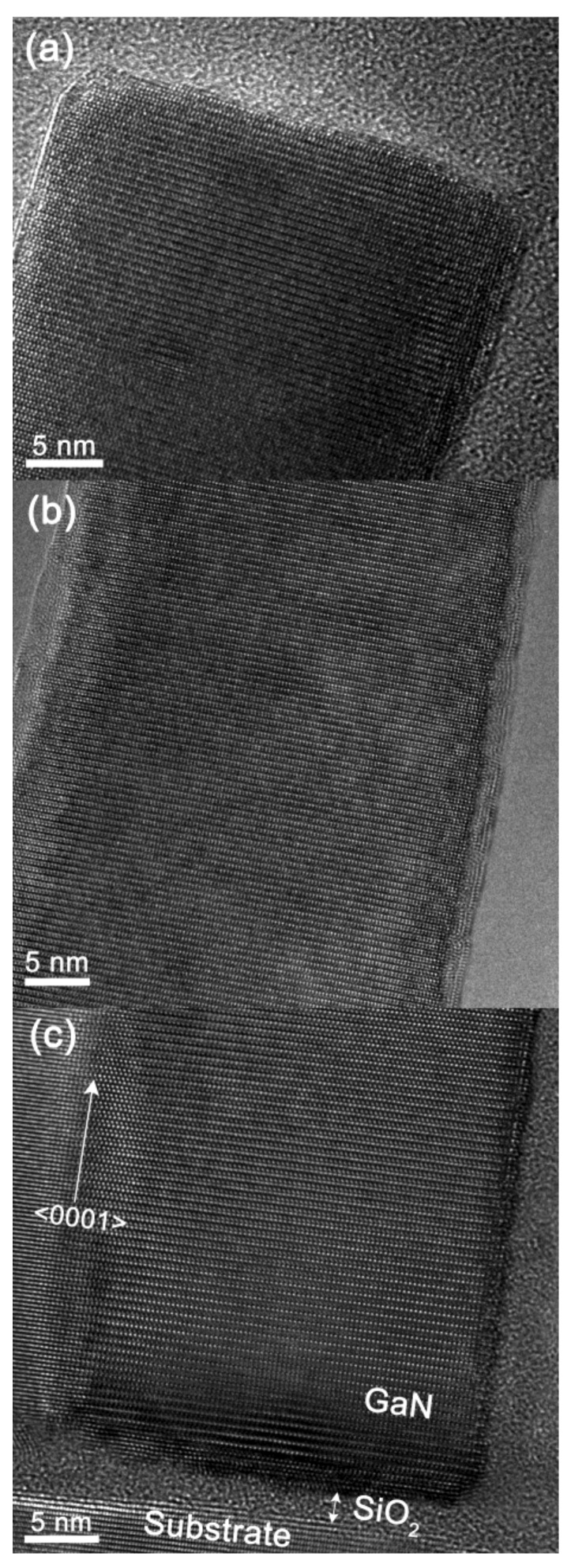
Cross-sectional HRTEM images from the GaN nanorod grown at *P*_N2_ = 2.5 mTorr on Si(111) substrate showing (**a**) the top part of the nanorod; (**b**) the middle part of the nanorod; and (**c**) the interface between the substrate and nanorods, containing the native oxide layer at the interface.

In order to study the structural quality of GaN-NRs, HRTEM was performed. For these studies, the two samples grown at *P*_N2_ = 4.5 mTorr and *P*_N2_ = 2.5 mTorr which demonstrated the highest aspect ratio and best optical properties, respectively, were selected. Figure 6 and Figure 7 show lattice resolved images from the interface, middle, and top part of the NRs grown at *P*_N2_ = 4.5 mTorr and *P*_N2_ = 2.5 mTorr, respectively. The HRTEM results showed that nucleation starts on the thin amorphous native oxide layer present on the Si (111) substrate surface for both *P*_N2_ = 4.5 mTorr and *P*_N2_ = 2.5 mTorr and that NRs have a random in-plane orientation, which is in agreement with the XRD texture results. The random in-plane orientation was expected, due to the amorphous surface oxide which lacks substrate lattice registry between the NRs and substrate. In both samples, the rods exhibit a high crystal quality from bottom to top. A few isolated stacking faults are seen randomly distributed along the growth direction in the *P*_N2_ = 4.5 mTorr sample, and the number of stacking faults is higher in this sample compared to the 2.5 mTorr sample in Figure 7, which exhibits no observed stacking faults. This corroborates the conclusion from the PL data that the *P*_N2_ = 2.5 mTorr sample is of exceptionally high structural quality. The observed stacking faults in Figure 6 may be related to the process dependent luminescence peak, visible at 3.429 eV in the 4 K µ-PL spectra of Figure 5. 

## 4. Conclusions 

By diluting the reactive N_2_ working gas with Ar in DC-MSE, single-crystal wurtzite GaN nanorods, grown onto Si (111) at a low total pressure of 5 mTorr, were achieved. This total working pressure is four times less than previously reported for growth in pure N_2_ environment, which will allow for better purity of the as-grown nanorods thanks to a lower probability of contamination from the residual gas partial pressure. The growth kinetics, structure quality, and optical properties of GaN are highly dependent on the ratio of Ar/N_2_. A process window in terms of Ar partial pressures was found, ranging from *P*_Ar_ = 0.5 mTorr to 2.5 mTorr, where GaN nanorods were formed. The highest aspect ratio of nanorods was achieved for *P*_Ar_ = 0.5 mTorr while the best optical properties were achieved at *P*_Ar_ = 2.5 mTorr, which is correlated to a reduction in structural defects, as evidenced by HRTEM for lower N_2_ pressures where a continuous film was achieved. The changes in nanorod morphology upon Ar-dilution of the N_2_ working gas are explained by a transition from N-rich growth conditions which promote a diffusion induced NR growth mode, to Ga-rich growth conditions; this growth behavior is in agreement with previous qualitative data on GaN NR growth by MBE. All samples exhibited strong band edge emissions located at around 3.48 eV, containing X_A_ and D^0^XA. At 4K PL. The peak located at 3.429 eV is associated with SF emission, which was evidenced by HRTEM. At *P*_N2_ = 2.5 mTorr, the Ga-target is close to a non-poisoned state which gives the most perfect crystal quality, which is reflected in exceptionally narrow BE emissions at 3.479 eV with a FWHM of only 1.7 meV and negligible SF emission. Such structural and optical properties are comparable to rods previously grown at three to four times higher total working pressures of pure N_2_.
